# A Prospective Study of Infectious Mononucleosis in College Students

**Published:** 2017-01-20

**Authors:** Leonard A. Jason, Ben Katz, Kristen Gleason, Stephanie McManimen, Madison Sunnquist, Taylor Thorpe

**Affiliations:** 1 DePaul University, Illinois, US; 2 Northwestern University Feinberg School of Medicine, Ann & Robert H Lurie Children’s Hospital of Chicago, US

**Keywords:** Chronic fatigue syndrome, Mononucleosis, Myalgic encephalomyelitis, Prospective study, Epstein barr virus, College students

## Abstract

**Background:**

The present study aims to prospectively investigate possible biological and psychological factors present in college students who will go on to develop chronic fatigue syndrome (CFS) following Infectious Mononucleosis (IM). Identification of risk factors predisposing patients towards developing CFS may help to understand the underlying mechanisms and ultimately prevent its occurrence. Our study is enrolling healthy college students over the age of 18. Enrollment began in March of 2013 and is ongoing.

**Methods:**

Biological and psychological data are collected when students are well (Stage 1), when they develop IM (Stage 2), and approximately 6 months after IM diagnosis (Stage 3).

**Results:**

Two case studies demonstrate the progression of student symptomology across all three stages.

**Conclusion:**

The Case Studies presented illustrate the usefulness of a prospective research design that tracks healthy students, following their trajectory of IM illness to either a) full recovery or b) diagnosis with CFS.

## Introduction

Epstein-Barr virus (EBV) is the most common cause of infectious mononucleosis (IM). EBV causes almost all cases of heterophile antibody positive IM, and the heterophile antibody test is positive in about 90% of young adults who develop IM [1]. In a recent review, Williams-Harmon, Jason, and Katz found that 1 – 5% of university students develop IM annually [2]. Studies also suggest that 9 – 12% of individuals go on to meet the criteria for chronic fatigue syndrome (CFS) 6 months following IM onset [3–6]. For example, White and colleagues [6] assessed patients 16–65 years of age with either glandular fever (the British term for IM) or an upper respiratory tract infection (URI) for the development of fatigue and/or CFS. Nine percent of subjects with glandular fever, whether due to EBV or a different etiologic agent, were fatigued and complained of excessive sleeping 6 months post-diagnosis, compared with none in the URI group. Similarly, Buchwald and colleagues [3] found that 12% of adults met criteria for CFS 6 months following IM. Likewise, Hickie and colleagues showed an 11% CFS rate 6 months following glandular fever or following two other similar systemic infections, Q fever and Ross River virus infection [4].

Several classic longitudinal studies have been conducted examining the relationship between the development of infection and problematic recovery trajectories. For example Imboden, Canter and Cluff performed a prospective study of influenza in which 600 employees in Fort Detrick were administered the Minnesota Multiphase Personality Inventory (MMPI) prior to the 1957 influenza season [7]. Twenty-six of the employees subsequently developed influenza. Fourteen recovered within 2 weeks, and 12 had symptoms for greater than 3 weeks (non-recovered). Results on the MMPI obtained prospectively, prior to illness, were significantly different between the recovered and non-recovered subjects. In another study by Kasl, Evans, and Niederman, military recruits were assessed both psychologically and serologically for the development of IM [8]. Of the 437 susceptible recruits, about one half became infected with EBV over the ensuing 4 years and about one quarter of infected recruits developed symptomatic IM. A specific psychological profile related to the cadets’ commitment to education and a military career, as well as their motivation, correlated with the development of symptomatic IM as well as the severity of the disease.

Studying younger subjects, Katz evaluated adolescents after developing IM (baseline) and at 6, 12 and 24 months following IM [5]. This study identified 39 adolescents (of the original sample of 801) diagnosed with CFS 6 months following a diagnosis of IM. In addition, 50 controls who were fully recovered from IM at baseline were also followed prospectively [9]. Those who were diagnosed with CFS as well as the recovered controls completed the Autonomic Symptom Checklist (ASC), which was adapted from The Autonomic Symptom Profile (ASP), and which had been validated for CFS against objective measures of autonomic function such as heart rate variability [10,11]. The differences between autonomic symptoms at baseline (approximately 2 months after the diagnosis of IM) and 6, 12, and 24 months following IM in CFS patients and recovered controls were evaluated. There was no difference between cases and recovered controls on sociodemographic, body weight, and activity variables. Nevertheless, the CFS group had significantly higher ASC scores even at baseline as well as 6, 12 and 24 months following IM [9].

Using the same cohort, Broderick retrospectively identified significant differences in IL-8 and IL-23 concentrations in the patient group at 24 months post-infection [12]. A network analysis showed that a particular profile of cytokines were expressed in a coordinated fashion and that levels of the IL-2, 6, 8 and 23 cytokines could be used to assign individuals to the patient or control group with an accuracy exceeding 80% when applied retrospectively relative to interferon gamma (IFN-γ) concentration. Overall these results indicate that subjects with post-IM CFS display a powerful pro-inflammatory cytokine profile. Using the same data set, Harvey retrospectively correlated lower ACTH levels at 6 months post-IM diagnosis with CFS; estradiol levels departed significantly from normal at 12 months only to recover at 24 months, and relative neutrophil count showed a significant departure from normal at 24 months in the CFS group [13].

Finally, Jason, Katz, and Shiraishi examined baseline variables that were gathered approximately two months following IM, including autonomic symptoms, days in bed since IM, perceived stress, stressful life events, difficulty functioning and attending school, family stress and psychiatric disorders [14]. Step-wise logistic regression findings indicated that baseline autonomic symptoms as well as days spent in bed since IM were the only significant predictors of those who met CFS criteria at 6 months. However, in none of these studies were subjects studied prior to the development of IM to assess for pre-illness predispositions towards developing CFS, and in many of them the data were analyzed retrospectively [3–6,9,12,13].

Because the pathophysiological underpinnings of the development of chronic fatigue syndrome (CFS) are still poorly understood, identification of risk factors predisposing to CFS should help uncover the underlying mechanisms of its genesis. In our ongoing longitudinal design, we are now following the trajectory of subjects from a healthy baseline status to either (a) IM and recovery or (b) IM followed by CFS. Our study has 3 stages. During Stage 1, we enroll otherwise healthy students. Stage 2 involves re-enrolling those students who develop IM. Finally, in Stage 3, we re-enroll students who progress to CFS following IM, as well as matched recovered controls. To demonstrate the differing trajectories between recovery and post-IM CFS, two illustrative case studies are presented.

## Methods

During Stage 1 of the project, we enroll otherwise healthy Northwestern University (NU) college students and use the Northwestern University Student Health Service (NUHS) to track their development of IM. An e-mail advertising the study is sent to all students at the beginning of each academic quarter. In addition, recruitment flyers are posted all around the NU campus, the health service center, and in students’ dorms. There are about 1,800 to 2,000 students in each matriculating class, and twenty-five percent of freshmen and 50% of sophomores utilize the NUHS center each year. As students wait in the health center, an advertisement for our study rotates among the electronic messages being broadcast routinely. After online consent, subjects complete several questionnaires on RED Cap using a personal computer or smart phone at their convenience [15].

These questionnaires include the DePaul Symptom Questionnaire (DSQ), the Medical Outcomes Study 36-Item Short-Form Health Survey (SF-36 or RAND Questionnaire), the Compass 31 autonomic symptom questionnaire, the Perceived Stress Scale, the Modifiable Activity Questionnaire, the Fatigue Severity Scale, the Coping Orientation to Problems Experienced Scale, and the Beck Depression and Anxiety Inventories [16–24]. Students then make an appointment at the NUHS where they sign a written consent and donate a small sample of blood, for which they are compensated. We obtain two tubes of peripheral blood in ethylene diamine tetra acetic acid (EDTA) tubes and serum separator tubes (SST) (20 ml) for storage on all subjects at each stage of the study.

In Stage 2, we enroll those students from Stage 1 who go on to develop heterophil (monopsot) - positive IM seen at the NUHS or at an unaffiliated physician. The students are re-consented, complete the same set of questionnaires and again provide a blood sample for which they are compensated. Five months after their original IM diagnosis, Stage 2 participants are contacted by phone and screened to determine if they are experiencing lingering symptoms following IM. All students deemed not recovered, and an equal number of recovered students (controls) matched by age, sex and class status when IM developed, are invited to participate in Stage 3 and are again consented. In addition to a third round of questionnaires and providing another blood sample, Stage 3 participants are given a free, comprehensive medical examination six months following their IM diagnosis by one of us (BZK) who is experienced with assessing CFS.

The physician screening evaluation includes an in-depth medical and psychiatric history, as well as general and neurological physical examination. Relevant medical information is gathered to exclude other possible medical causes of chronic fatigue per the Fukuda criteria and as refined by Reeves [25,26]. The histories of all symptoms related to CFS are also gathered. Laboratory tests deemed necessary to rule out other illnesses are also be obtained [5,25]. This study was approved by the Institutional Review Boards of Northwestern University, DePaul University and the Ann & Robert H Lurie Children’s Hospital of Chicago.

## Case Definitions

At each stage a standardized instrument, the DePaul Symptom Questionnaire (DSQ), is used to assess whether participants meet case definitions. The DSQ is an updated self-report measure of CFS symptomatology and illness history and includes items that assess the dimensions of various case definitions, including the Fukuda CFS criteria, the Canadian Clinical ME and CFS criteria, and the ME International Consensus Criteria [25,27,28]. The DSQ has 54 items assessing symptoms along with 6 additional questions required for case definition classification. Participants are asked to indicate if their fatigue has been present for 6 months or longer and if their symptoms began before the onset of their fatigue or IM. Participants are also asked to rate the frequency and severity for each of the 54 symptoms assessed.

### Fukuda criteria

To be diagnosed using the Fukuda criteria, participants need to experience persistent or relapsing fatigue for a period of six or more months concurrent with at least four of eight somatic symptoms that do not predate the fatigue, including sore throat, lymph node pain, muscle pain, joint pain, post-exertion malaise, headaches of a new or different type, memory and concentration difficulties, and un refreshing sleep [25]. Participants also need to experience substantial reductions in occupational, educational or personal activities as a result of their illness. The substantial reduction criterion as established by Jason is measured by determining if participants score at or below at least two of the three following subscale cutoffs on the SF-36 [29]: Role Physical score ≤ 50, Social Functioning score ≤ 62.5, and Vitality score ≤ 35.

### Canadian criteria

The Canadian criteria case definition is modeled after the Canadian clinical case definition [27]. Participants need to have unexplained, persistent or relapsing chronic fatigue over the past 6 months that was not the result of ongoing exertion and was not substantially alleviated by rest. Participants must have experienced substantial reduction in previous levels of educational, social and personal activities. Substantial reduction in activity is again measured using the SF-36 cutoffs described above. Participants also need to have the following symptoms: post-exertional malaise, unrefreshing sleep or disturbance of sleep quantity, pain (myofascial, joint, abdominal and/or head pain), and two or more neurocognitive manifestations such as impairment of memory and short term memory consolidation. Additionally, they must have at least one symptom from two of the following three categories: autonomic (e.g., neurally mediated hypotension, postural orthostatic tachycardia), neuroendocrine (e.g., recurrent feelings of feverishness and cold extremities, subnormal body temperature), or immunologic (e.g., recurrent flu-like symptoms, non-exudative sore or scratchy throat). Frequency and severity ratings of a 2 or higher, indicating the symptom occurs at least half of the time and is of moderate or greater severity, are needed as well.

### IOM criteria

The IOM clinical criteria were operationalized by having participants meet the following four criteria: 1) Substantial reductions in functioning, as described above; 2) Post-exertion malaise as manifested by: soreness after mild activity, drained/sick after mild activity, minimum exercise makes tired, muscle weakness, dead/heavy feeling after exercise, and mentally tired after the slightest effort; 3) Sleep dysfunction, which can include unrefreshing sleep, problems staying asleep, problems falling asleep, waking up early, and needing to nap daily; and 4) Neurocognitive impairment or orthostatic intolerance. Neurocognitive impairment includes: Difficulty paying attention, difficulty expressing thoughts, problems remembering, and absent-mindedness, only being able to focus on one thing at a time, slowness of thought, difficulty understanding, and difficulty paying attention. Orthostatic intolerance is defined as dizziness or fainting, shortness of breath, unsteadiness, irregular heartbeat, or chest pain. The symptoms needed to occur at least half of the time with at least moderate severity to be considered present.

Each participant’s DSQ and SF-36 results from the baseline, IM, and 6-month follow-up questionnaires are evaluated to determine whether or not they meet one or more of the three case definitions outlined above. Additionally, as a secondary indicator of reduced functioning, results from the Modifiable Activity Questionnaire are also examined [20]. The following two case studies present the differing IM illness trajectories of a patient who developed CFS following IM versus a recovered control participant, focusing on whether symptom patterns at each stage of the study time point warrant a diagnosis of CFS as evaluated using the three different sets of case definition criteria described above.

## Results

### Participant A

**Figure 1:**
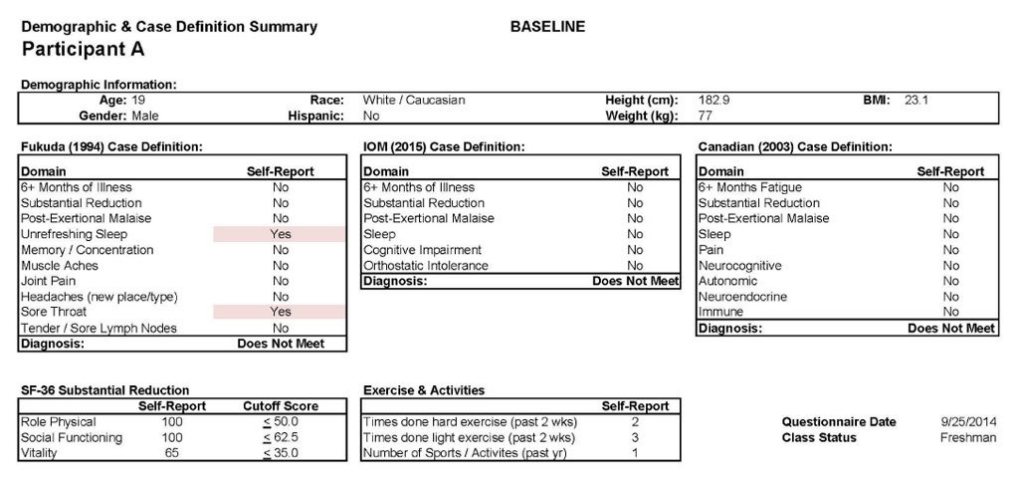
Healthy baseline (Stage 1) data for Participant A.

This individual recovered from IM. Figure 1 shows baseline (Stage 1) data for this participant: He was a 19 year old Caucasian/White male. The DSQ results show that this participant did not meet any of the case definitions for CFS/IM (the Fukuda criteria, the Canadian ME and CFS criteria, or the more recent IOM criteria) at baseline [25,27,30].

**Figure 2:**
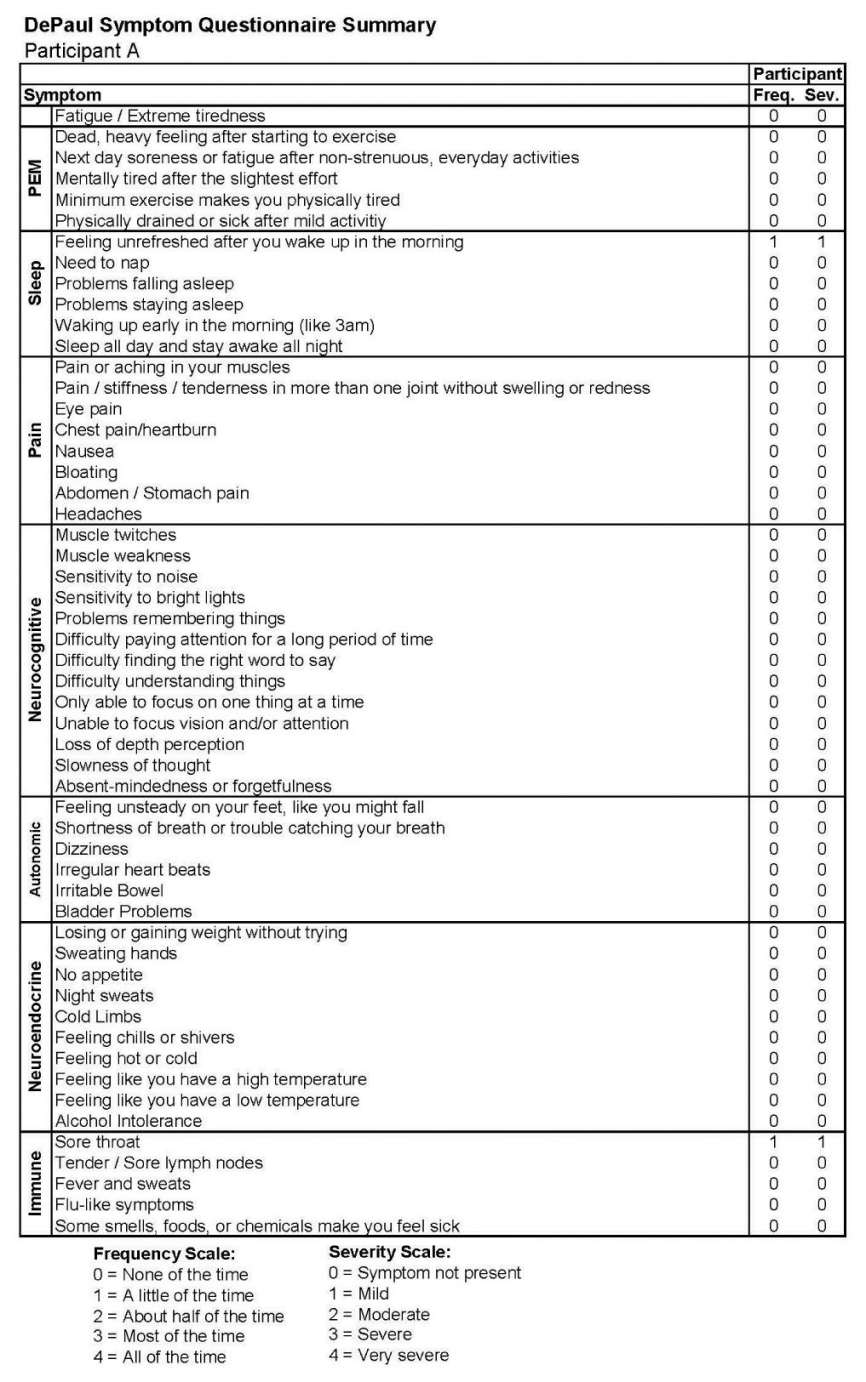
Healthy baseline symptom data (Stage 1) for Participant A.

Figure 1 also includes data regarding the SF-36 as well as the number of activities reported by the participant, both of which indicate high levels of activity and involvement. Figure 2 provides more detailed data from the baseline DSQ for this control participant, and as is evident, no symptom occurred at least half of the time (with a rating of 2 or greater) with at least moderate severity (with a rating of 2 or greater). The frequency and severity scales are shown at the bottom of the figure.

**Figure 3:**
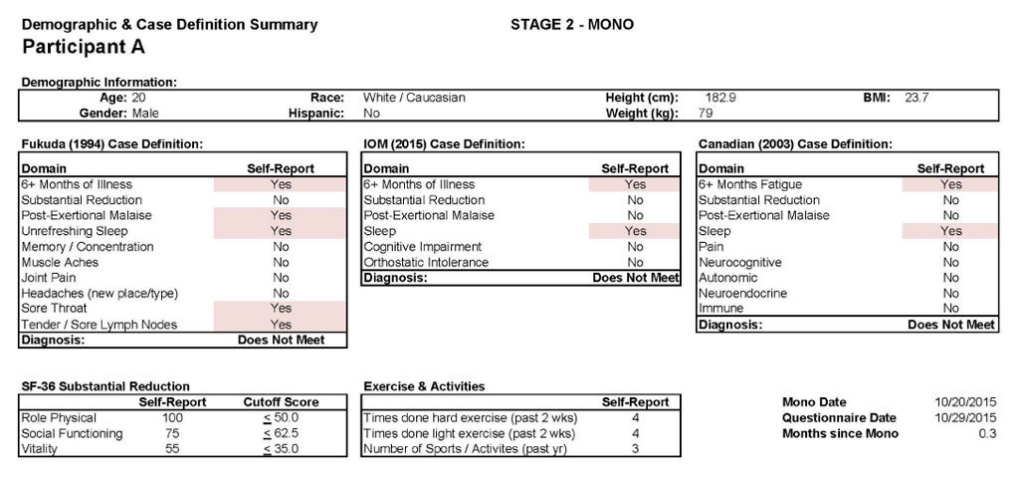
Stage 2 IM data for Participant A.

**Figure 4:**
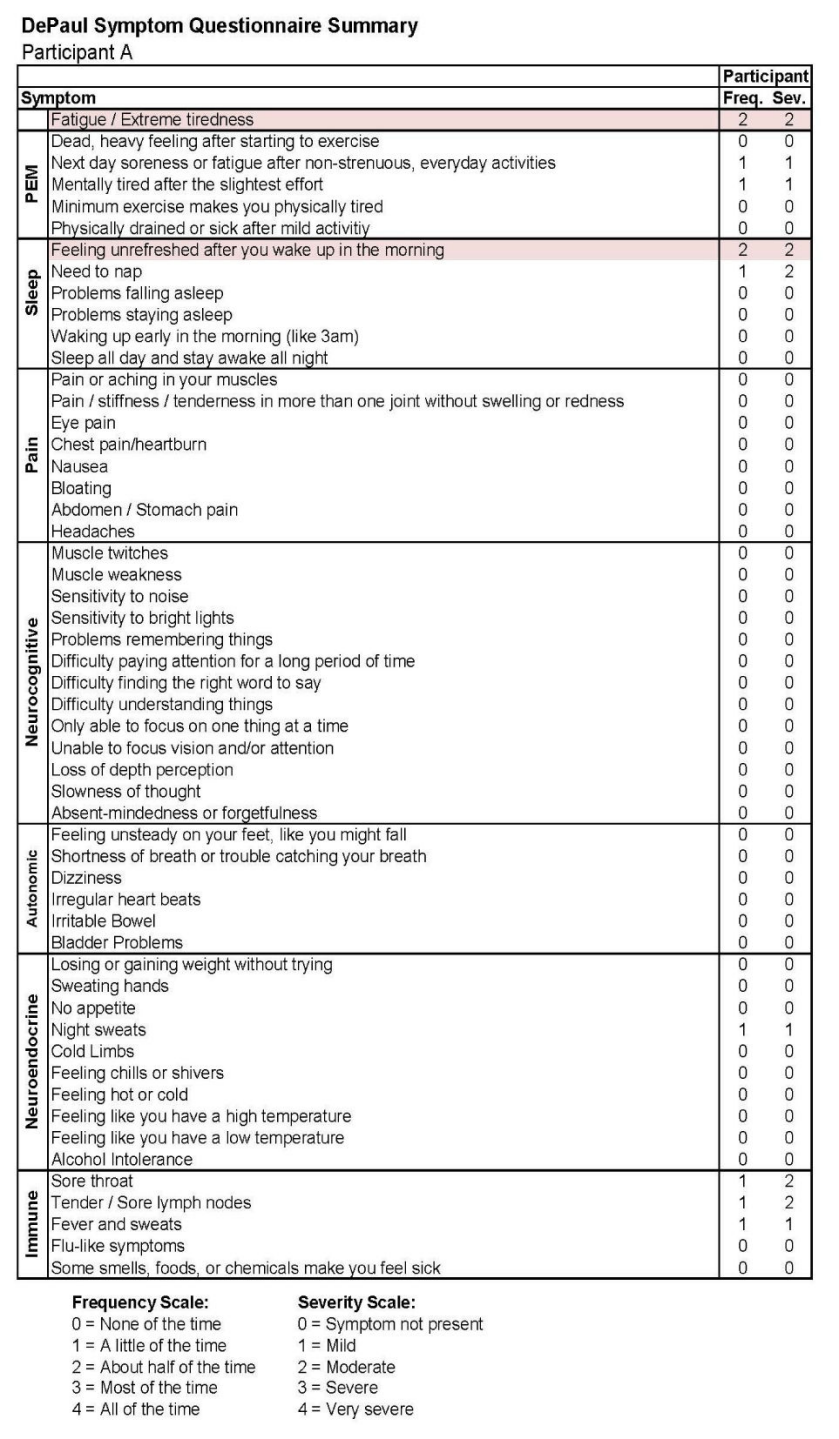
Stage 2 IM symptom data for Participant A.

**Figure 5:**
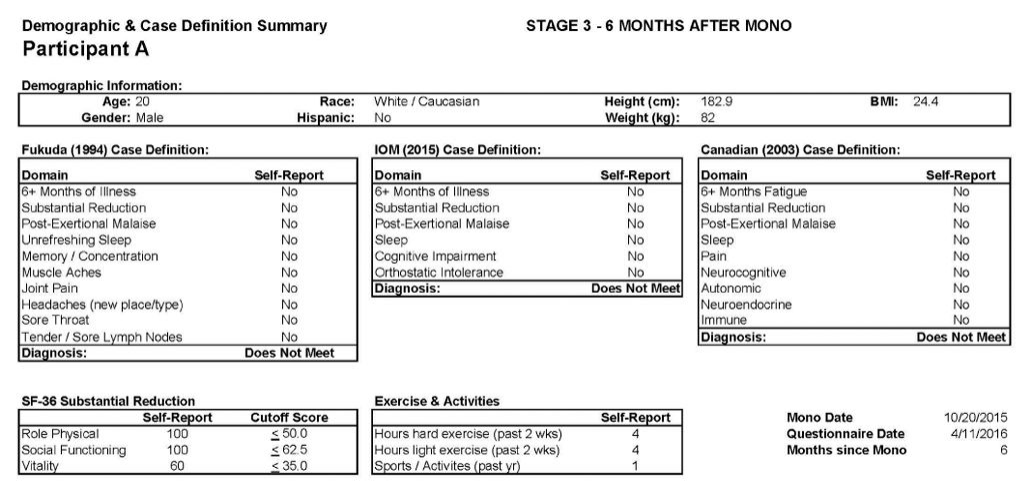
Stage 3 data for Participant A.

**Figure 6:**
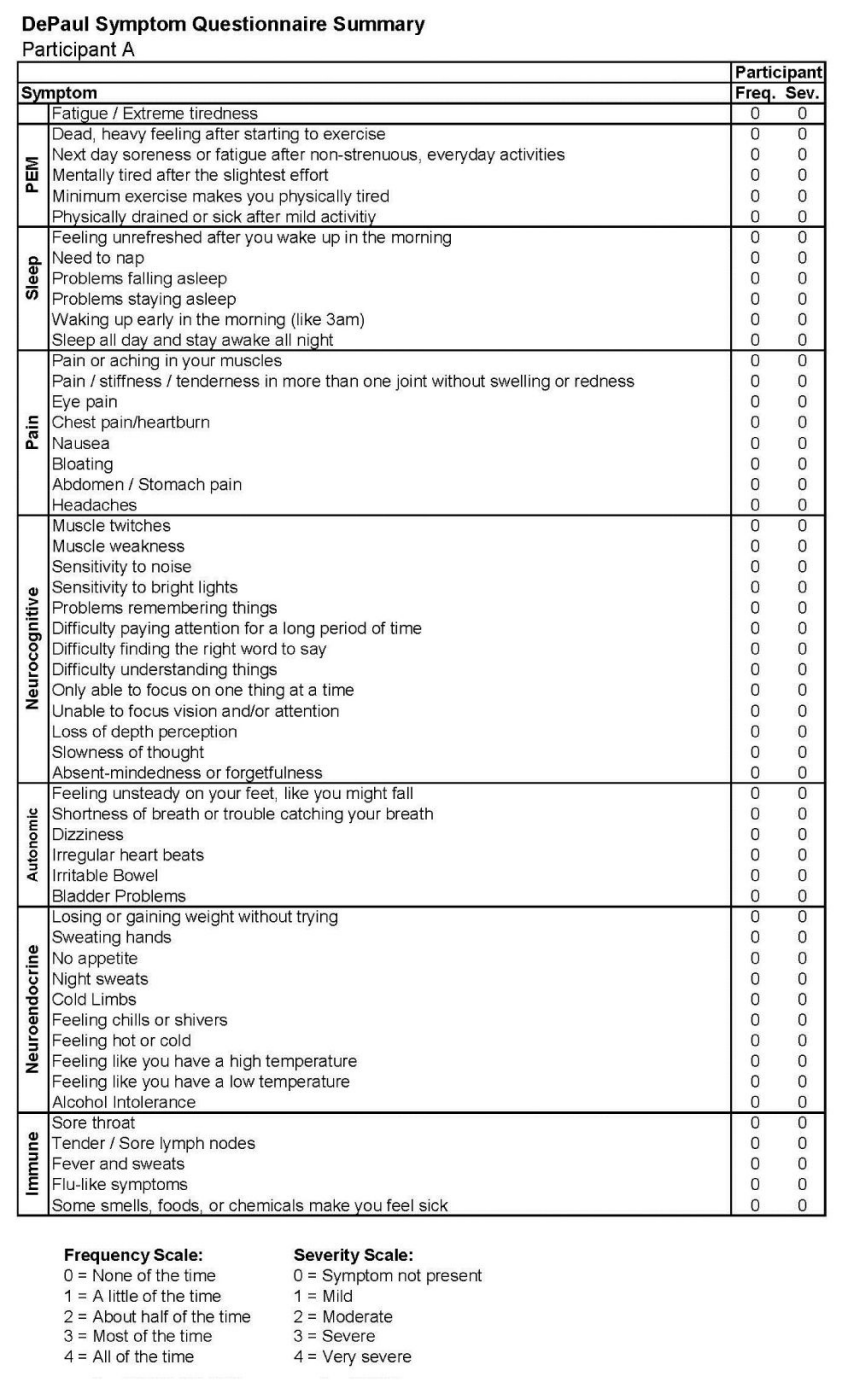
Stage 3 symptom data for Participant A.

Figures 3 and 4 report the DSQ results taken shortly (0.3 months) after the participant’s IM diagnosis (Stage 2) and indicate that even at this time he continued to be quite active. In the more detailed report in Figure 4, there are two symptoms that are shaded, suggesting some sleep and fatigue issues, which likely reflected the IM. Figures 5 and 6 provide comparable data 6 months later (Stage 3), and again indicate that the individual did not meet any of the CFS case definitions and that symptoms had generally been reduced from the prior IM assessment.

### Participant B

**Figure 7:**
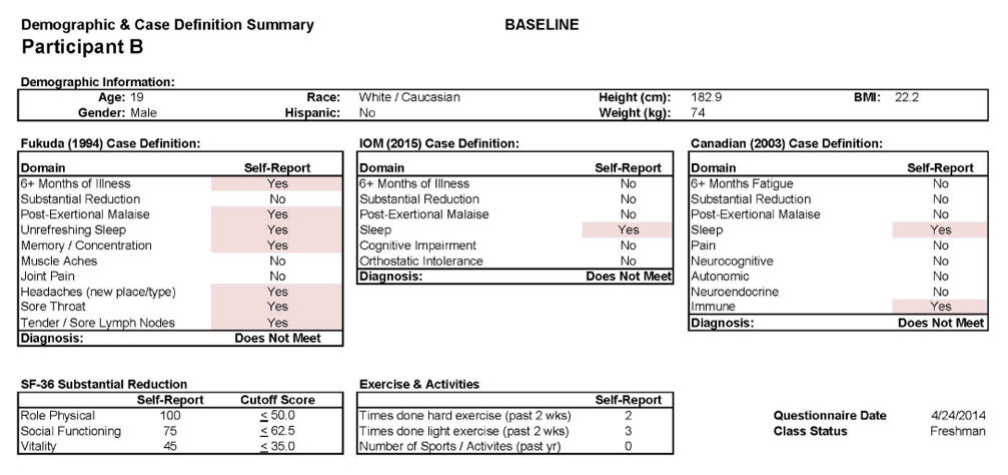
Healthy baseline (Stage 1) data for Participant B.

**Figure 8:**
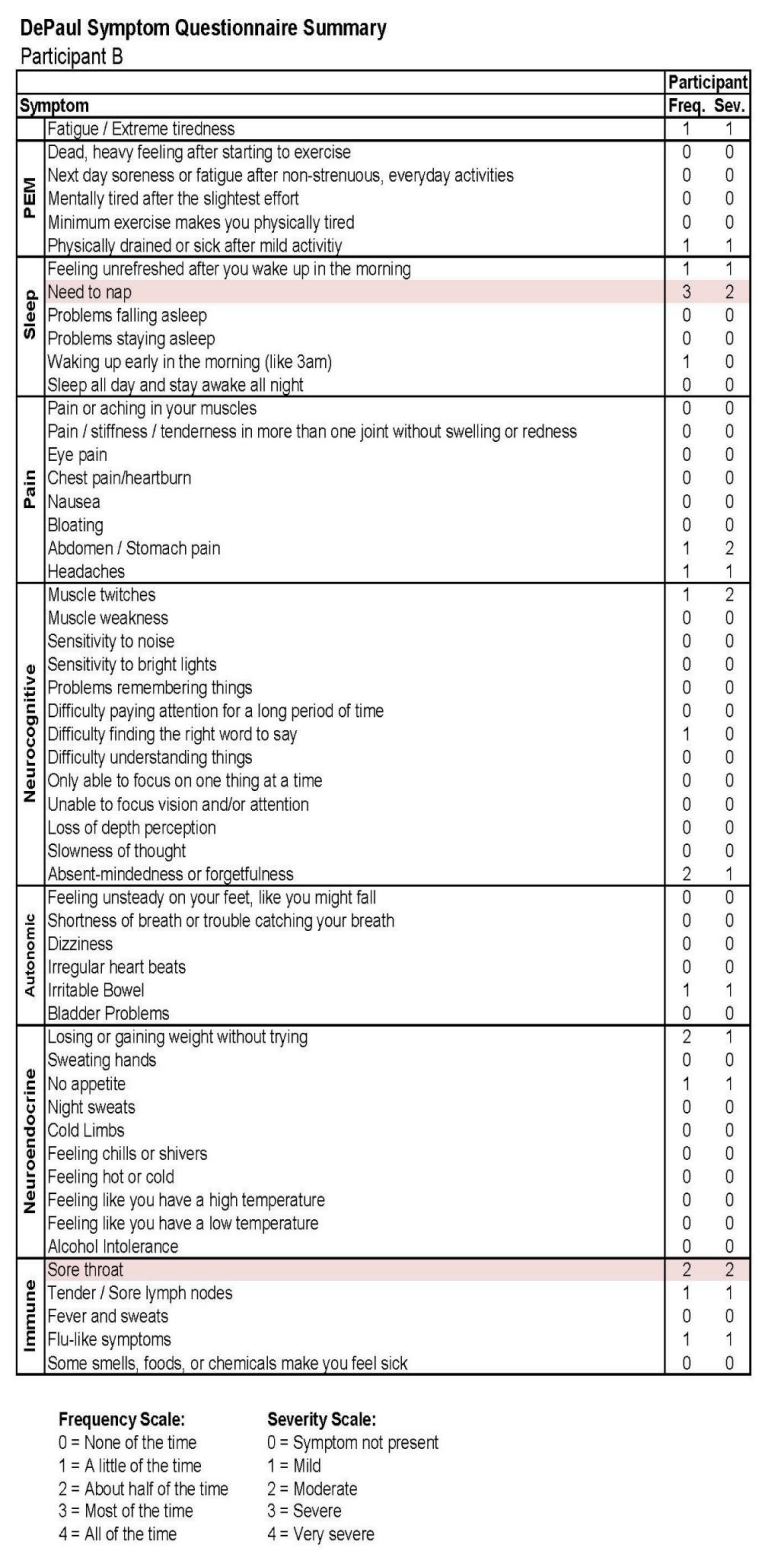
Healthy baseline (Stage 1) symptom data for Participant B.

This individual developed CFS following IM. As with Participant A, in Figure 7, we see baseline (Stage 1) demographic information, indicating a matched 19 year old Caucasian/White male. As expected, at baseline no CFS case definition was met using any of the aforementioned criteria. Figure 8 provides more detailed data from the DSQ at baseline and shows only a few symptoms (need to nap and sore throat) are present occurring at least half the time (with a rating of 2 or greater) with at least moderate severity (with a rating of 2 or greater).

**Figure 9:**
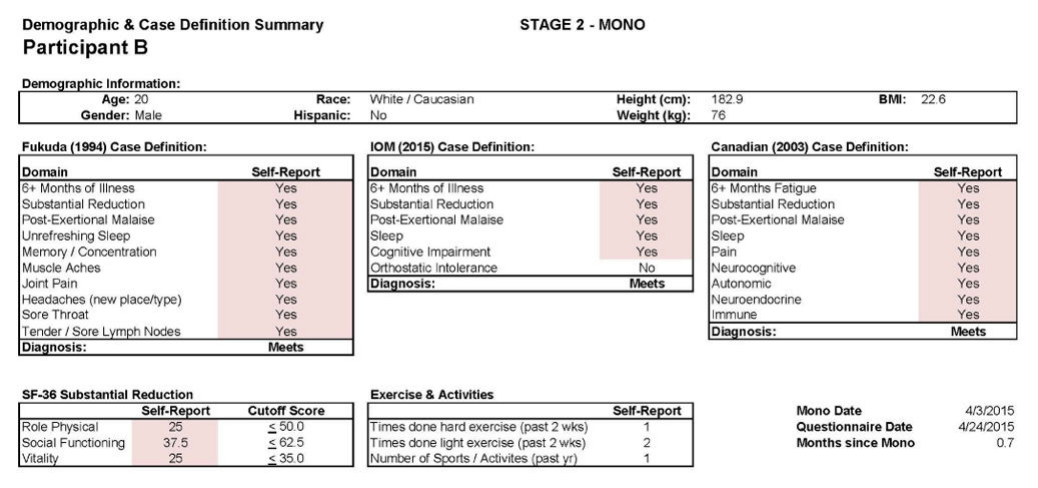
Stage 2 IM data for Participant B.

**Figure 10:**
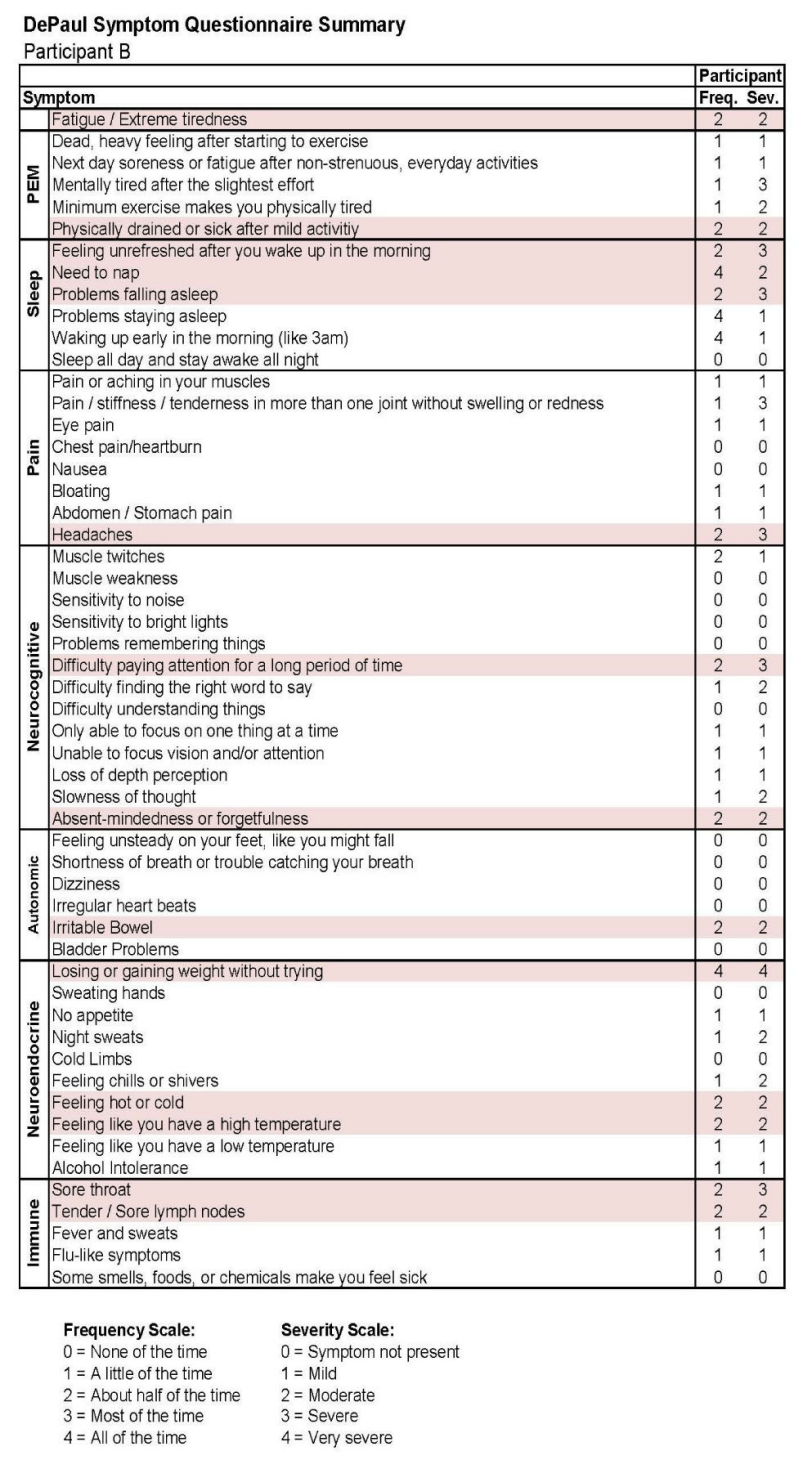
Stage 2 IM symptom data for Participant B.

**Figure 11:**
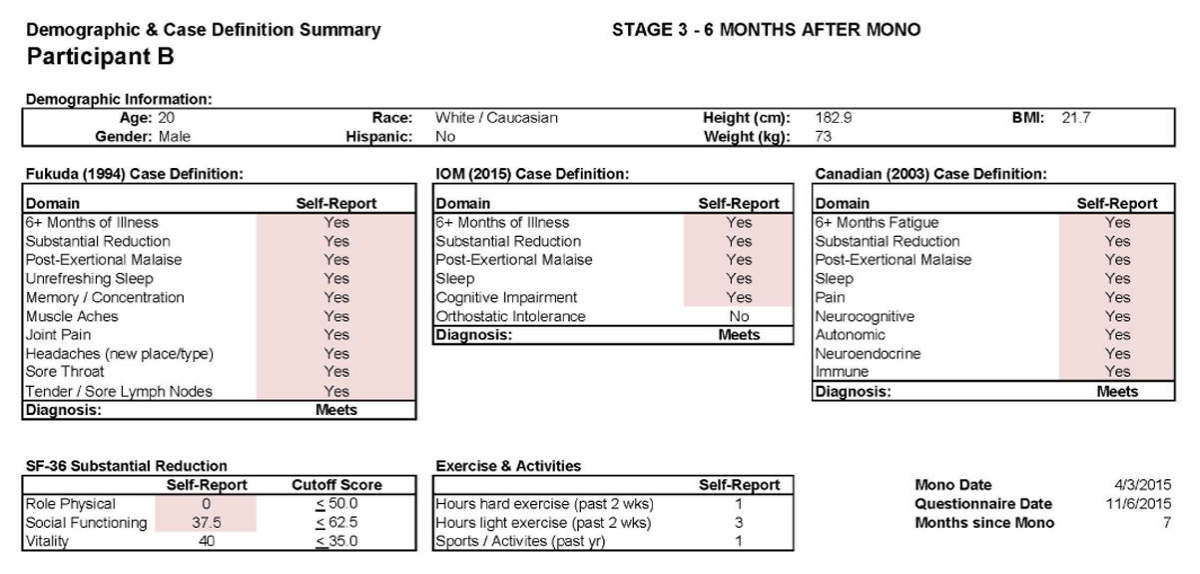
Stage 3 data for Participant B.

**Figure 12:**
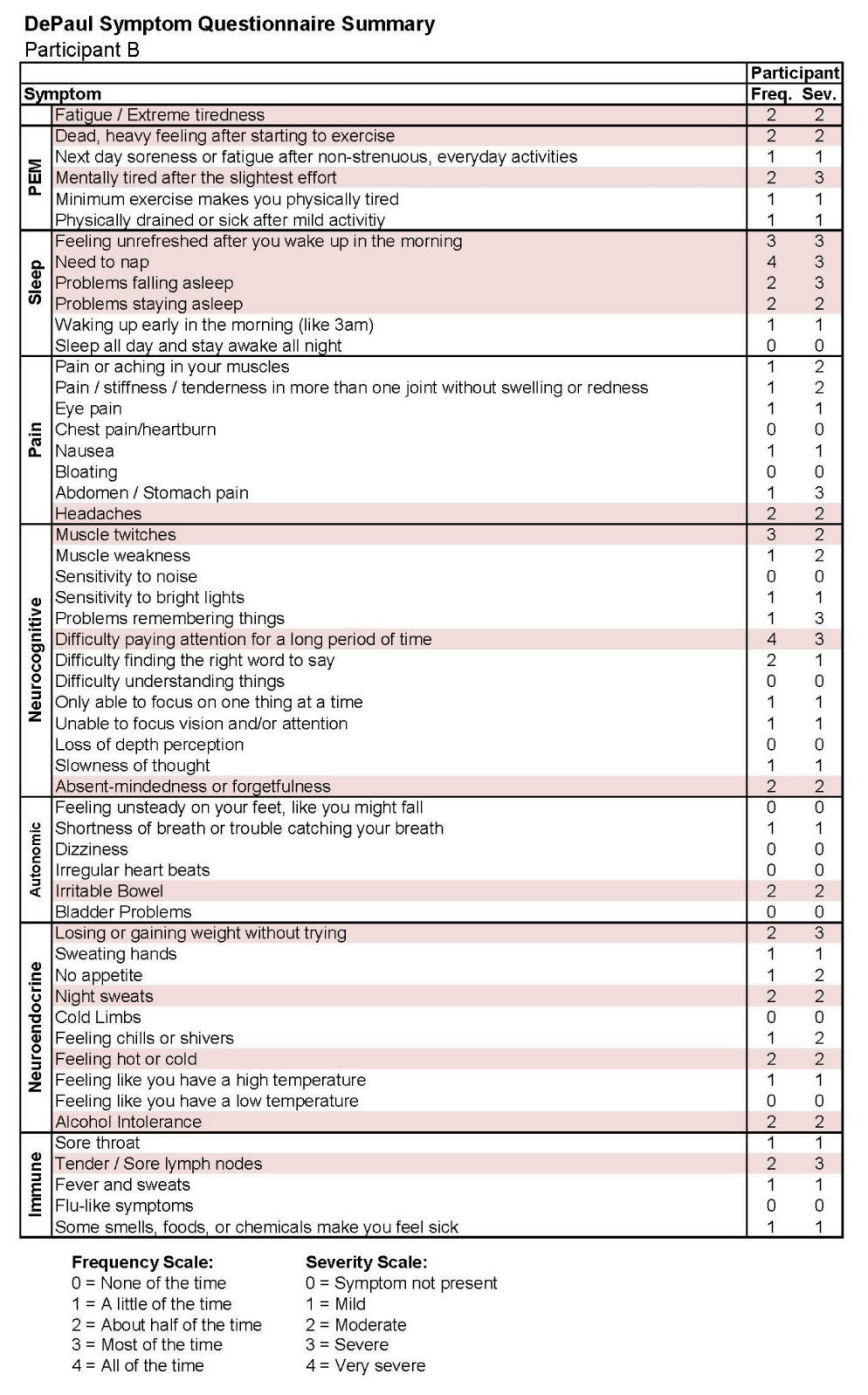
Stage 3 symptom data for Participant B.

Figures 9 and 10 report what this participant experienced following the diagnosis of IM (Stage 2), and indicate that the participant was quite symptomatic. In fact, at that time he had a sufficient number of symptoms to meet all three case definitions of CFS and there were substantial reductions in activities. This was also the case 6 months later, as seen in Figures 11 and 12. This individual thus had a severe case of IM and did not recover within 6 months.

### Comment

Approximately 1 – 5% of college students develop IM every year, and about 12% of young adults who develop IM will have lingering symptoms, such as tiredness, and will meet criteria for CFS [2, 5]. The main purpose of this prospective study is to determine which biological and/or psychological factors are present in young adults that may predict who will go on to develop CFS following IM.

In order to do so, we are studying Northwestern University (NU) undergraduate students. Those who participate and later develop IM are then evaluated 6 months later to assess if they recover or go on to develop CFS. The longitudinal design is notable, following the trajectory of subjects from a baseline healthy status to either (a) IM and complete recovery or (b) IM followed by CFS. The participant pool of college students helps assure study retention. The case studies described are preliminary results from the successful tracking of IM illness trajectories in a college population. They demonstrate both an ability to distinguish between uncomplicated and complicated recovery from IM as well as the usefulness of a prospective research design. This study is unique in that it has been able to collect data on participants prior to the development of IM and later CFS.

## Limitations

The results presented here are preliminary case examples, and as such can only illustrate potential illness trajectories. More information is needed to determine the examples presented above are indicative of broader patterns of illness and recovery in our sample. As further results become available other aspects of the participant illness trajectories will help shed more light on the pathophysiology of CFS. For example, we are obtaining pre-illness plasma in order to determine if there is a cytokine profile predictive for developing CFS following IM, as has been suggested previously [12].

## Conclusions

The pathophysiologic underpinnings of the development of CFS are poorly understood, and identification of risk factors predisposing patients towards developing these conditions should help us understand the underlying mechanisms involved. The a priori defined study of these variables may yield a pre-morbid diathesis for development of CFS. In addition, the identification of such outcomes could significantly alter the therapeutic strategies for infectious mononucleosis.
